# Prediction of neonatal survival among Pacific Islander preterm births in the US

**DOI:** 10.1371/journal.pone.0316048

**Published:** 2024-12-31

**Authors:** Bohao Wu, Sarah Taylor, Veronika Shabanova, Nicola L. Hawley

**Affiliations:** 1 Department of Chronic Disease Epidemiology, Yale University School of Public Health, New Haven, Connecticut, United States of America; 2 Division of Neonatal-Perinatal Medicine, Yale University School of Medicine, New Haven, Connecticut, United States of America; 3 Department of Pediatrics, Yale University School of Medicine, New Haven, Connecticut, United States of America; 4 Department of Biostatistics, Yale University School of Medicine, New Haven, Connecticut, United States of America; University of Cambridge, UNITED KINGDOM OF GREAT BRITAIN AND NORTHERN IRELAND

## Abstract

**Objective:**

Predicting neonatal survival is essential for targeting interventions to reduce neonatal mortality. Pacific Islanders have been underrepresented in existing prediction tools and have unique, maternal obesity-related risk factors for both preterm birth and neonatal mortality. Using neonatal sex, birth weight, and gestational age, we developed a graphical tool for neonatal survival among Pacific Islander singletons in the United States.

**Methods:**

Birth-infant death data files from the United States National Center for Health Statistics were used (2014–2018). Pacific Islander mothers and singletons without congenital anomalies born between 22–36 gestational weeks were included. Poisson regression models were used to predict neonatal mortality (<28 days of life) rate including neonatal sex, birth weight, and gestational age in weeks as predictors. Predicted survival rates in the graphical tool were calculated as "1 minus mortality rate”.

**Results:**

Of the 5192 included neonates, the neonatal mortality rate was 2.0%; 43.5% of mothers had pre-pregnancy obesity, and 16.5% of neonates were born large-for-gestational age. Birth weight and gestational age had a non-linear association with neonatal death, and their interaction was included in the model. Retaining neonatal sex, models with gestational age at birth or both birth weight and gestational age at birth performed better than the model with birth weight only.

**Conclusion:**

This is the first graphical tool for neonatal survival prediction among preterm-born Pacific Islander singletons in the United States. Using only neonatal sex, birth weight, and gestational age, this graphical tool is a straightforward reference for survival among groups of neonates with similar characteristics.

## Introduction

Neonatal mortality (death within the first 28 days of life [1]) in the US declined from 4.1 per 1 000 live births in 2011 to 3.7 in 2019 (most recently available data from March of Dimes) [2]. However, during the same period, the prevalence of preterm birth (PTB, live birth at <37 gestational weeks [3]) increased from 9.8% to 10.2% [4], and the prevalence of low birth weight (LBW) increased from 8.1% to 8.3% [5]. Gestational age (GA) and birth weight (BW) are two important indicators for neonatal mortality and have been used in the development of tools to predict neonatal survival [6–8]. Although not used as a basis for individual care decisions, prediction of neonatal survival rate for groups of infants with certain characteristics may be informative for clinicians treating populations at high risk. These prediction modelling outcomes can provide population-based estimates of neonatal survival rate to further support clinicians’ assessments [9]. Graphical prognosis tools, including the original ‘Draper Grid’, are most commonly used for clinical reference, given their simple visualization of expected sex-specific survival based on gestational week and birth weight [6, 10, 11].

Racial disparities in infant mortality [2] (2017–2019: Black, 10.8 per 1 000 live births; White, 4.6 per 1 000 live births), preterm birth [4] (2018–2020: Black, 14.2%; White, 9.2%), and LBW [5] (2018–2020: Black, 13.8%; White, 7.0%) are evident in the US. One group currently underrepresented in perinatal research are Pacific Islanders. Data on the health of Pacific Islanders in the US is sparse, and frequent aggregation with Asian Americans exacerbates their underrepresentation. To our knowledge, no prognosis tool has either included a significant proportion of Pacific Islanders in their reference population, nor has a tool been specifically developed for this group. Current neonatal survival rate prediction tools have generally been designed for specific populations, like for Canadians, Australians, or for residents in the United Kingdom, for example [6, 7, 11–13]. Those tools do not transport well across populations due to different population characteristics and clinical practice variation [7, 14]. Based on trends observed in other populations, the unique health profile of Pacific Islanders—a high prevalence of overweight/obesity and obesity-related non-communicable diseases [15, 16] before and during pregnancy—is expected to place them at high risk of both preterm birth and neonatal death [17, 18]. Given their underrepresentation and frequent aggregation with other groups, understanding of the BW by GA distribution among Pacific Islanders is also limited, with studies reporting both higher prevalence of macrosomia among term births compared to other ethnic groups, and also greater incidence of low birth weight [19]. Here we aimed to develop a graphical tool for the prediction of survival rate among Pacific Islanders born between 22–36 weeks gestation in the US using neonatal sex, BW, and GA.

## Materials and methods

**Fig 1:**
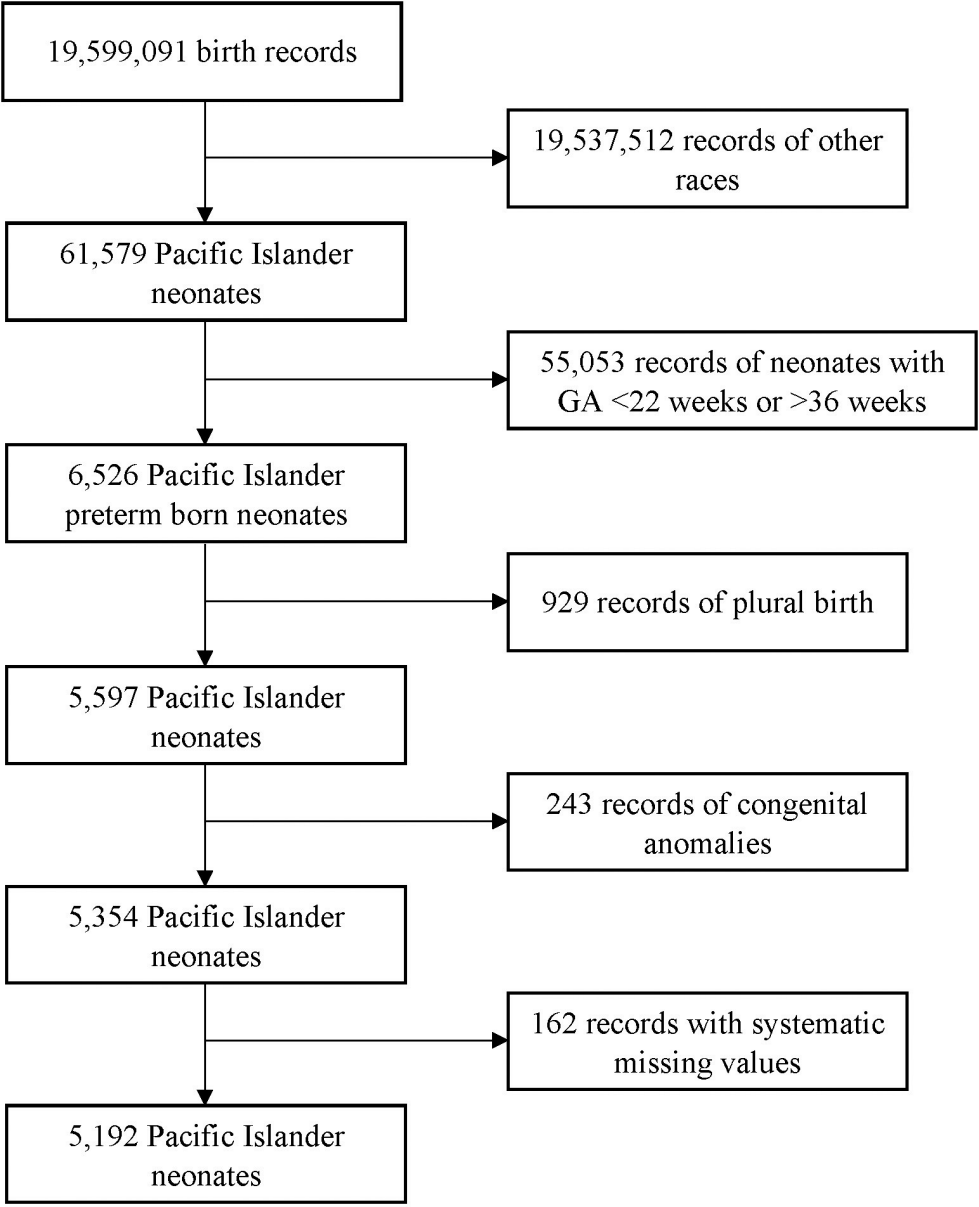
Flowchart of the sample selection from the 2014–2018 birth cohort birth-infant death data files. GA, gestational age.

We used 2014–2018 cohort linked birth-infant death data files (corresponding to deaths that occurred between 2014–2019) from the US National Center for Health Statistics (NCHS) [20]. Our study population included preterm-born neonates (22–36 gestational weeks) whose mothers identified as Native Hawaiian or Other Pacific Islander. Plural births, neonates with congenital anomalies, and blank records with maternal race only were excluded. In total, data from n = 5192 neonates were included for the prediction of survival (**Fig 1**).

### Exposure

Demographic characteristics of included mother-neonate dyads were obtained from birth and death certificates. Individual characteristics included GA (measured by obstetric estimate [the best estimate based on ultrasound-confirmed LMP, LMP (in the absence of early ultrasound] or early ultrasound [in the absence of LMP)]) at birth, degree of prematurity (extreme PTB 22–27 weeks, very PTB 28–31 weeks, moderate to late PTB 32–36 weeks) [3], BW and BW classification (I: LBW <2500 g, normal BW 2500–4000 g, macrosomia >4000 g [21]; II: small-for-GA [SGA], appropriate-for-GA [AGA], and large-for-GA [LGA], based on the 2017 US BW percentiles for singletons [22]), and neonatal sex at birth. Maternal characteristics included ethnicity (Hawaiian, Guamanian, Samoan, Other Pacific Islander), age (<20 years, 20–34 years, > = 35 years), pre-pregnancy body mass index (BMI, underweight <18.5, normal 18.5–24.9, overweight 25.0–29.9, obesity I 30.0–34.9, obesity II 35.0–39.9, extreme obesity III > = 40.0). In order to ensure the largest available sample size for analyses, missing values for pre-pregnancy BMI (5.3%) were imputed with maximum likelihood via expectation maximization (EM; one imputed dataset) [23].

### Outcomes

Our primary outcome is neonatal mortality which is defined as death within the first 28 days of life [1]. Predicted survival rates in the graphical tool were based on "1 minus mortality rate”. Other birth outcomes including five-minute Appearance, Pulse, Grimace, Activity and Respiration (APGAR) score [24] and final route and method of birth were also reported to characterize the study population.

### Statistical analysis

We first described the general preterm birth prevalence among all Pacific Islander neonates and neonatal mortality rate among Pacific Islander preterm singletons without congenital anomalies in the US 2014–2018 birth cohort. To characterize the study population, we then examined neonatal sex-distributed basic demographic characteristics, five-minute APGAR score and final route and method of birth. Data were presented as numbers and percentages for categorical variables, and as medians with lower (Q1) and upper (Q3) quartiles for non-normally distributed continuous variables. Differences by neonatal sex were examined using *x*^2^ tests or Fisher’s exact tests for categorical variables and with exact Wilcoxon two-sample tests for continuous variables. Monte Carlo estimations of exact P-values were presented when continuous variables were not normally distributed [25]. We also described the frequency distribution of included neonates by both BW (in increments of 250 g) and GA (in increments of 1 week) stratified by sex (**S1 Fig**).

Poisson regression models (link = log) were used to predict the neonatal mortality rate using neonatal sex at birth, BW, and GA in weeks as predictors. We tested their independent effects, as well as their statistical interactions (retained in the model at a two-sided α of 0.10). The final three models, adjusted by neonatal sex, were BW-based only, GA-based only, and BW with GA and their interaction. In the context of risk prediction, any multicollinearity between BW and GA would not influence the mortality rate predictions and their precision or the goodness-of-fit statistics [26]. The model fit of competing models was assessed by Akaike Information Criterion (AIC) [27]. Due to the curve-linear association between log-scaled mortality rate and predictors BW and GA, the latter two were transformed to the square root form prior to being used in the regressions in order to preserve the linear combination of regression parameters in the models. Overdispersion was assessed by Pearson Chi-square / degree of freedom (>2 indicating overdispersion [28]); the scaling parameter was set to 1 when overdispersion occurred. The plot of the observed moving sum of residuals against the predicted values assessed the functional form of our regression models [29], with a large probability indicating a correctly specified model for the mean response. Predictive accuracy was evaluated using Receiver Operating Characteristic (ROC) curves [30] which measure how well the model discriminates between neonates with or without mortality (>0.80 indicating a good discrimination), as well as with decile calibration plots [31] which measure how well the predicted probabilities of mortality agree with the observed probabilities of mortality. The internal validity of the models was assessed using bootstrapping with 500-samples with replacement [32]. Predicted neonatal survival rates were calculated from “1 minus neonatal mortality rate” and were presented as Draper Grids [10]. The presented predicted survival rate in each cell is at the midpoint of the cell (week of GA plus 0.5 week and BW midpoint). For ease of interpretation, the US 3^rd^, 10^th^, 25^th^, 50^th^, 75^th^, 90^th^, 97^th^ BW percentiles for GA [22] were added to the plot. Analyses were performed using R software (version 3.6.2) [33], RStudio [34] and SAS software version 9.4 (SAS Institute Inc.). Per Yale University institutional review board policy, no research ethics board approval was required for the use of this deidentified, public data.

## Results

A total of 5192 preterm-born (22–36 gestational weeks) Pacific Islander neonates without congenital anomalies were included in survival rate prediction models, including 2428 females (46.8%) and 2764 males (53.2%). The averaged preterm birth prevalence among the study population was 9.3%. Within the study population, the neonatal mortality rate among preterm-born singletons without congenital anomalies was 20.8 per 1 000 preterm births.

Length of gestation and BW were not observed to be different between female and male Pacific Islander neonates (**Table 1**). Among female neonates, 7.5% were SGA and 17.8% were LGA, and among male neonates, 8.5% were SGA and 15.3% were LGA, using the 2017 US reference data for singletons [22], which indicated that Pacific Islander neonates had a relatively larger BW compared to US neonates of other racial/ethnic groups. A more detailed distribution of the study population by BW and GA stratified by sex is presented in **S1 Fig**. Most Pacific Islander mothers identified as ‘Other’ Pacific Islanders (>60.0%). Adolescent mothers were 6.9% of all included mothers, and the percentage of mothers with advanced age (> = 35 years) was 20.0%. Of the included mothers, 29.6% had a pre-pregnancy BMI indicative of overweight, and 43.5% had obesity. Most preterm-born neonates included in the study population had a five-minute APGAR score of 9–10. The majority were born via spontaneous vaginal birth (55.8%), although Caesarean births accounted for 41.8% of all births.

**Table 1:** Characteristics of Included Pacific Islander mother-neonate dyads in the US, 2014–2018 birth cohort.

Characteristics		Female (n = 2 428)		Male (n = 2 764)		P-value
		N or median	% or Q1-Q3	N or median	% or Q1-Q3	
Neonatal death		41	1.7	65	2.4	0.09
Gestational age (median, Q1-Q3, week)		34.1	32.2–36.0	34	32.1–35.9	0.21
Preterm birth categories						
	Extreme PTB (22–27 weeks)	134	5.5	133	4.8	
	Very PTB (28–31 weeks)	184	7.6	240	8.7	
	Moderate to Late PTB (32–36 weeks)	2110	86.9	2391	86.5	0.2
Birth weight categories (I, median, Q1-Q3, g)		2456.9	1951.5–2962.4	2482.6	1983.8–2981.4	0.1
	Low birth weight (<2500 g)	1146	47.2	1276	46.2	
	Normal weight (2500–4000 g)	1241	51.1	1446	52.3	
	Macrosomia (>4000 g)	41	1.7	42	1.5	0.64
Birth weight categories (II)						
	Small for gestational age	181	7.5	235	8.5	
	Appropriate for gestational age	1814	74.7	2105	76.2	
	Large for gestational age	433	17.8	424	15.3	0.03
Maternal ethnicity						
	Hawaiian	241	9.9	254	9.2	
	Guamanian	283	11.7	307	11.1	
	Samoan	403	16.6	498	18	
	Other Pacific Islanders	1501	61.8	1705	61.7	0.46
Maternal age (median, Q1-Q3, year)		28.6	24.3–32.8	28.6	24.2–32.9	0.95
	<20 years	165	6.8	194	7	
	20–34 years	1785	73.5	2011	72.8	
	> = 35 years	478	19.7	559	20.2	0.83
Maternal pre-pregnancy BMI (median, Q1-Q3, km/m^2^)		29.7	24.9–34.5	29.8	24.9–34.7	0.81
	Underweight (<18.5)	64	2.6	66	2.4	
	Normal (18.5–24.9)	586	24.1	680	24.6	
	Overweight (25.0–29.9)	730	30.1	806	29.2	
	Obesity I (30.0–34.9)	557	22.9	627	22.7	
	Obesity II (35.0–39.9)	282	11.6	333	12.1	
	Extreme Obesity III (> = 40.0)	209	8.6	252	9.1	0.92
Five-minute APGAR score^a^						
	A score of 0–3	55	2.3	69	2.5	
	A score of 4–6	131	5.5	185	6.7	
	A score of 7–8	689	28.7	736	26.8	
	A score of 9–10	1529	63.6	1758	64	0.15
Final route and method of birth^a^						
	Spontaneous	1369	56.4	1530	55.4	
	Forceps	16	0.7	24	0.9	
	Vacuum	27	1.1	53	1.9	
	Caesarean	1015	41.8	1157	41.9	0.09

Q1, lower quartile; Q3, upper quartile; BMI, body mass index; GWG, gestational weight gain.

^a^ Missing value for labelled variables: five-minute APGAR score (n = 40), final route and method of birth (n = 1).

Parameter estimates, model fit statistics, and model prediction statistics are presented for three neonate sex-adjusted competing models: GA-based, BW-based, and BW-GA-based models (**Table 2**). For descriptions of models where additional interactions were tested, see **S1 File**. The value of Pearson Chi-square/DF indicates that overdispersion was evident in the BW-based model, but not in the GA-based and BW-GA models. Comparing the AIC values, the GA-based and BW-GA-based models had the smallest values, indicating their better fit to the data than the BW-based model (**Table 3**).

**Table 2:** Poisson regression models for neonatal mortality rate among selected Pacific Islander neonates in the 2014–2018 US birth cohort.

Model	Formula	Pearson Chi-square/DF
GA-based model	$log(mortality)=\beta_{0}+\beta_{1}Female+\beta_{2}\sqrt{GA}$	1.007
BW-based model^a^	$log(mortality)=\beta_{0}+{\beta_{1}Female+\beta }_{2}\sqrt{BW}$	2.799
BW-GA-based model	$log(mortality)=\beta_{0}+{\beta_{1}Female+\beta }_{2}\sqrt{GA}+\beta_{3}\sqrt{BW}+\beta_{4}\sqrt{GA}\times \sqrt{BW}$	0.920

GA, gestational age. BW, birth weight. DF, degree of freedom.

^a^ Scaling parameter for labelled models was set to 1 due to overdispersion (Pearson Chi-square/DF>2).

**Table 3:** Parameter estimates, model fit, and predicted accuracy of Poisson regression models for neonatal mortality rate among selected Pacific Islander neonates in the 2014–2018 US birth cohort.

Model	Coef	Est	SE	95% CI		P-value	AIC	Non-bootstrapped		Bootstrapped	
								ROC-AUC	ROC-AUC 95% CI	ROC-AUC	ROC-AUC 95% CI
GA-based model	*β*_0_	19.827	1.175	17.525	22.129	<0.001	676.524	0.897	0.859–0.935	0.897	0.895–0.899
	*β*_1_	-0.274	0.200	-0.665	0.117	0.170					
	*β*_2_	-4.258	0.228	-4.704	-3.811	<0.001					
BW-based model^a^	*β*_0_	3.168	0.581	2.029	4.307	<0.001	715.439	0.879	0.834–0.923	0.879	0.877–0.882
	*β*_1_	-0.413	0.334	-1.067	0.241	0.216					
	*β*_2_	-0.168	0.017	-0.201	-0.135	<0.001					
BW-GA- based model	*β*_0_	26.125	5.083	16.164	36.087	<0.001	676.927	0.895	0.856–0.935	0.896	0.893–0.898
	*β*_1_	-0.295	0.201	-0.689	0.098	0.141					
	*β*_2_	-5.238	1.011	-7.219	-3.256	<0.001					
	*β*_3_	-0.307	0.167	-0.634	0.020	0.066					
	*β*_*4*_	0.051	0.029	-0.006	0.107	0.078					

Coef, coefficient. Est, estimate. SE, standard error. 95% CI, 95% confidence interval. AIC, Akaike information criterion. ROC-AUC, area under the Receiver Operating Characteristic curve. GA, gestational age. BW, birth weight. DF, degree of freedom.

^a^ Scaling parameter for labelled models was set to 1 due to overdispersion (Pearson Chi-square/DF>2).

**Fig 2:**
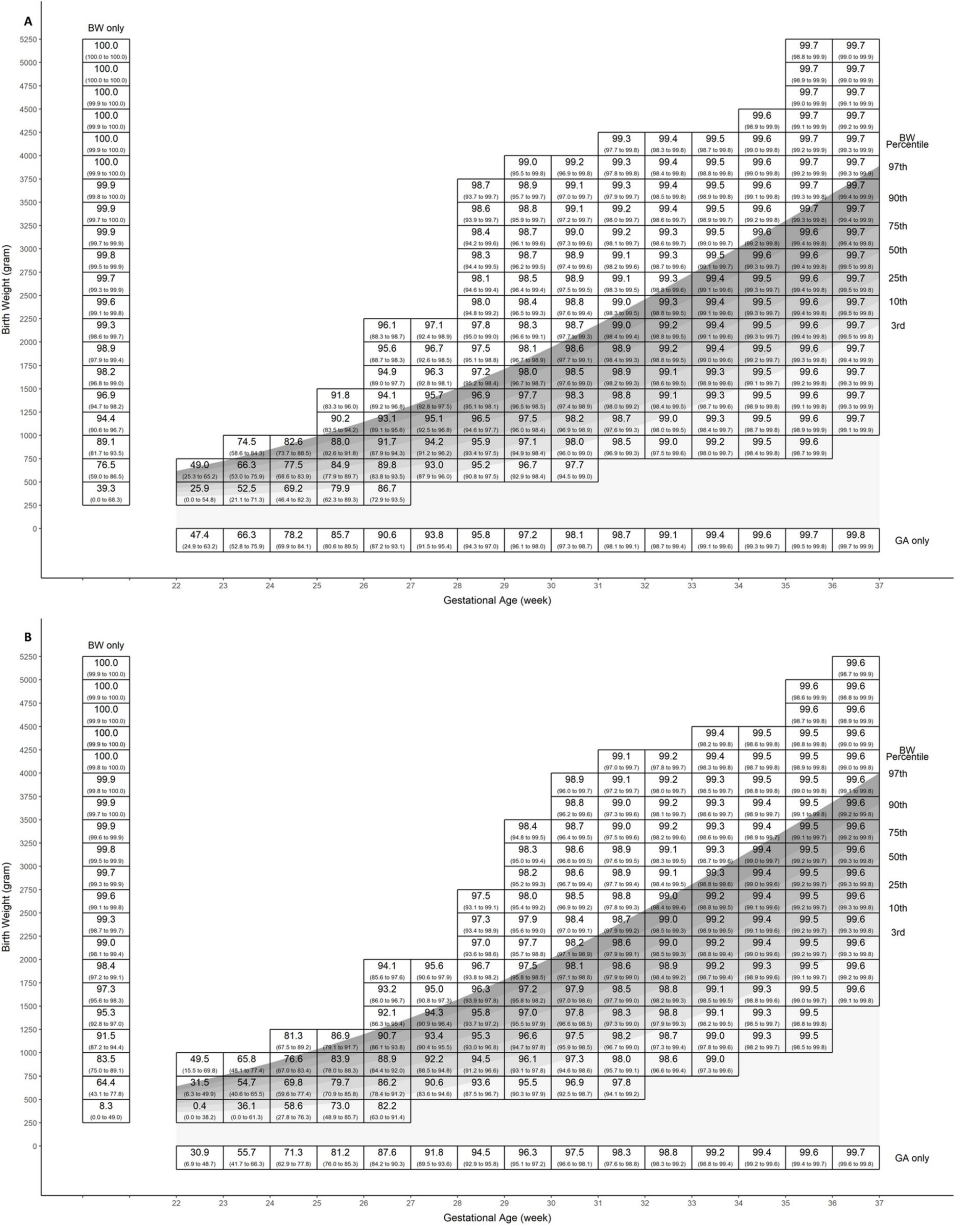
Grid of predicted neonatal survival rate according to sex, birth weight, and gestational age (BW-GA-based model). The point estimate in each cell is the predicted survival rate at the midpoint of the cell. The numbers in parentheses are lower and upper limit of predicted survival rate within each cell. A, female plot. B, male plot. BW, birth weight; GA, gestational age.

The observed moving sum of residuals plot for BW-GA-based model (p = 0.82) depicted a random pattern indicating that the log-link for the mean response as well as the selected covariates adequately captured neonatal mortality, but not so for the BW-based (p = 0.001) and GA-based (p = 0.09) models (**S2 Fig**). For the ROC value, all three models had a value >0.80 indicating a good discrimination, which was also confirmed in the bootstrapped sample (**Table 3**), with the highest performance by GA-based and BW-GA-based models. For the decile calibration plot (**S3 and S4 Figs**), using the non-bootstrapped sample, all three models had a wider 95% confidence interval (CI) of the agreement between the predicted and observed probabilities at higher probabilities of mortality, but in both the non-bootstrapped and the bootstrapped samples, the BW-GA-based model had the best calibration. Predicted survival rates from the BW-GA-based model are presented in **Fig 2** stratified by neonatal sex. Female neonates had a relatively higher survival rate compared to male neonates, although the 95% confidence intervals overlapped given the same BW and GA.

## Discussion

To our knowledge, this is the first study to predict neonatal survival rate among Pacific Islanders in the US. Using obstetric estimate as the GA measurement method, the averaged preterm birth prevalence for singletons without congenital anomalies was 9.3% among Pacific Islander neonates from the 2014–2018 birth cohort in the US, which is higher than the prevalence among the general US population (7.6% to 8.1% using the same dataset, 2014–2018 with the same GA measurement method) [35]. Within our Pacific Islander population, the neonatal mortality rate among preterm-born singletons without congenital anomalies was 20.8 per 1 000 preterm births, which is similar to the rate among comparable White preterm-born singletons (19.2 per 1 000 preterm births) in the US [35]. As we hypothesized, the prevalence of pre-pregnancy obesity (BMI > = 30) among Pacific Islander women (43.5%) was higher than among non-Hispanic White (39.8%) women in the US [36]. Likely as an associated complication, Pacific Islander neonates also had a relatively larger BW compared to neonates of other races based on a large proportion of the study population being born LGA (16.5%).

Among Pacific Islander singletons without congenital anomalies, GA was the most important predictor of neonatal death. When using BW without GA, the increased value of AIC, the decreased value of ROC-AUC (for non-bootstrapped and bootstrapped samples), as well as a systematic trend on the observed moving sum of residuals, indicated the poorer performance of BW alone in predicting neonatal survival among infants born to Pacific Islander women. This is consistent with another study, conducted in Australia and New Zealand, which also reported a decreased discriminatory power when BW was included in the model for neonatal mortality [7]. This is to be expected given the heterogeneity in weight present at any given week of gestation. While the GA model has the best fit (smallest AIC), the model that also incorporates BW and their interaction shows better calibration while maintaining the same discriminatory power, indicating the value of birthweight when used in combination with GA. Even though neonatal sex did not reach the statistical threshold for the alpha-level, it was still retained in our model due to different birth weight for gestational age at birth [22] and neonatal mortality rate [37] by sex. Furthermore, it is plausible that with additional years of data from NCHS, a larger sample size will confirm the clinical importance of neonatal sex on neonatal survival and clear the statistical threshold in this population of infants.

We did not attempt to compare the performance of the model developed here to previously published tools that describe mortality or survival among other populations [6, 7, 10–13]. Several previous studies [7, 14] suggest that such tools do not transport well across populations due to differences in population characteristics (such as the level of obesity among Pacific Islanders), clinical practice variation, diagnosis and case mix differences, and chance variation. This further underlines the importance of population-based specific tools for neonatal survival prediction.

Several limitations should be noted. Like any other model, our predictions of neonatal survival should not be used for diagnosis in any individual neonate. Meanwhile, the small sample size of extreme preterm births (n = 267) included in our study population may also influence our accuracy at the earliest gestational ages. Plural births and neonates with congenital anomalies were not included and are at a much higher risk of neonatal death compared to singletons or neonates without congenital anomalies [38]. Given our sample size, we did not have sufficient power to develop survival predictions for these groups and efforts should be made to repeat this analyses with additional years of data, building sufficient sample size to inform estimates for this group. Fetal growth among plural births is much more complicated and discordant growth may occur and influence perinatal outcomes [39]. Death due to congenital anomalies usually occurs very early in gestation [40], and may be recorded as a stillbirth or termination of pregnancy, both of which were not included in the linked infant datafiles.

The major strength of our study is that we used only three simple predictors for developing this prediction plot. Similar graphical neonatal death prognosis tools have been used widely in other populations [6, 10, 11] indicating the accuracy of using these three predictors only. Presenting our estimates in the style of Draper Grid is straight-forward and serves as a reference for healthcare providers conducting parent consultations [10, 11] or for health policy makers [41, 42]. Moreover, we used the most recent national-level data for Pacific Islanders in the US with GA measured by obstetric estimate, which increased the reliability of our results [43].

## Conclusions

Pacific Islander mothers have a relatively higher prevalence of preterm birth, neonatal mortality rate, pre-pregnancy obesity, and LGA compared to White mothers in the US. To address underrepresentation of this group, and considering their unique risks for preterm birth, we developed a race-specific graphical tool to predict neonatal survival for Pacific Islander singletons without congenital anomalies born preterm in the US. With additional validation, this graphical tool may serve as a straight-forward reference to support discussions and decisions by clinicians or health policy makers.

## Supporting information

S1 File (DOCX)

S1 Fig Frequency distribution of included neonates in the US 2014–2018 birth cohort by sex. A, female plot. B, male plot. BW, birth weight; GA, gestational age.
(TIF)

S2 Fig Observed moving sum of residuals plots for GA-based, BW-based, and BW-GA-based model for neonatal mortality rates among selected Pacific Islander neonates in the 2014–2018 US birth cohort. A, GA-based model. B, BW-based model. C, BW-GA-based model. BW, birth weight; GA, gestational age. All models are adjusted by neonatal sex.
(TIF)

S3 Fig Decile calibration plot for non-bootstrapped data, models for GA-based, BW-based, and BW-GA-based model for neonatal mortality rates among selected Pacific Islander neonates in the 2014–2018 US birth cohort. A, GA-based model. B, BW-based model. C, BW-GA-based model. BW, birth weight; GA, gestational age. All models are adjusted by neonatal sex.
(TIF)

S4 Fig Decile calibration plot for bootstrapped data, models for GA-only, BW-only, and BW-GA model for neonatal mortality rates among selected Pacific Islander neonates in the 2014–2018 US birth cohort. A, GA-based model. B, BW-based model. C, BW-GA-based model. BW, birth weight; GA, gestational age. All models are adjusted by neonatal sex.
(TIF)

S5 Fig Observed moving sum of residuals plots for other explored Poisson regression models for neonatal mortality rates among selected Pacific Islander neonates in the 2014–2018 US birth cohort. A, Model 1. B, Model 2. C, Model 3. D, Model 4. E, Model 5.
(TIF)

S6 Fig Decile calibration plot for non-bootstrapped data, other explored Poisson regression models for neonatal mortality rates among selected Pacific Islander neonates in the 2014–2018 US birth cohort. A, Model 1. B, Model 2. C, Model 3. D, Model 4. E, Model 5.
(TIF)

S7 Fig Decile calibration plot for bootstrapped data, other explored Poisson regression models for neonatal mortality rates among selected Pacific Islander neonates in the 2014–2018 US birth cohort. A, Model 1. B, Model 2. C, Model 3. D, Model 4. E, Model 5.
(TIF)

## Abbreviations

APGAR: Appearance, Pulse, Grimace, Activity and Respiration
AGA: Appropriate for gestational age
AIC: Akaike Information Criterion
BMI: Body mass index
BW: Birth weight
DF: Degree of freedom
GA: Gestational age
LGA: Large for gestational age
LBW: Low birth weight
Q1: Lower quartile
ROC: Receiver Operating Characteristic
SGA: Small for gestational age
US: United States
Q3: Upper quartile
WHO: World Health Organization
