# Supplementary material for: Prediction of neonatal survival among Pacific Islander preterm births in the US
Source: PLoS One. 2024 Dec 31;19(12):e0316048. doi: 10.1371/journal.pone.0316048 (PMC11687717; doi:10.1371/journal.pone.0316048)
Supplement: S1 File — (DOCX) [file pone.0316048.s001.docx]

**Supplementary Materials**

**List of Supplementary Tables**

**S1 Table** Other explored Poisson regression models for neonatal mortality rate among selected Pacific Islander neonates in the 2014-2018 US birth cohort.

| **Model** | **Formula** | **Pearson Chi-square/DF** |
| --- | --- | --- |
| Model 1 | $log(mortality)=\beta_{0}+\beta_{1}Female+\beta_{2}\sqrt{GA}+\beta_{3}Female\times\sqrt{GA}$ | 1.055 |
| Model 2^a^ | $log(mortality)=\beta_{0}+{\beta_{1}Female+\beta}_{2}\sqrt{BW}+\beta_{3}Female\times\sqrt{BW}$ | 3.301 |
| Model 3 | $log\left( mortality \right)=\beta_{0}+{\beta_{1}Female+\beta}_{2}\sqrt{GA}+\beta_{3}\sqrt{BW}+\beta_{4}\sqrt{GA}\times\sqrt{BW}+\beta_{5}Female\times\sqrt{GA}$ | 0.962 |
| Model 4 | $log\left( mortality \right)=\beta_{0}+{\beta_{1}Female+\beta}_{2}\sqrt{GA}+\beta_{3}\sqrt{BW}+\beta_{4}\sqrt{GA}\times\sqrt{BW}+\beta_{5}Female\times\sqrt{BW}$ | 0.945 |
| Model 5 | $log\left( mortality \right)=\beta_{0}+{\beta_{1}Female+\beta}_{2}\sqrt{GA}+\beta_{3}\sqrt{BW}+\beta_{4}\sqrt{GA}\times\sqrt{BW}+\beta_{5}Female\times\sqrt{GA}+\beta_{6}Female\times\sqrt{BW}$ | 0.947 |

GA, gestational age. BW, birth weight. DF, degree of freedom.

^a^ Scaling parameter for labeled models was set to 1 due to overdispersion (Pearson Chi-square/DF>2).

**S2 Table** Parameter estimates, model fit, and predicted accuracy of other explored Poisson regression models for neonatal mortality rate among selected Pacific Islander neonates in the 2014-2018 US birth cohort.

| **Model** | **Coef** | **Est** | **SE** | **95% CI** | | **P-value** | **AIC** | **Non-bootstrapped** | | **Bootstrapped** | |
| --- | --- | --- | --- | --- | --- | --- | --- | --- | --- | --- | --- |
|  |  |  |  |  |  |  |  | **ROC-AUC** | **ROC-AUC**  **95% CI** | **ROC-AUC** | **ROC-AUC**  **95% CI** |
| Model 1 | $\beta_{0}$ | 18.630 | 1.451 | 15.787 | 21.474 | <.001 | 676.666 | 0.895 | 0.856-0.9341 | 0.895 | 0.893-0.898 |
|  | $\beta_{1}$ | 3.078 | 2.495 | -1.812 | 7.969 | 0.217 |  |  |  |  |  |
|  | $\beta_{2}$ | -4.026 | 0.279 | -4.573 | -3.478 | <.001 |  |  |  |  |  |
|  | $\beta_{3}$ | -0.653 | 0.486 | -1.606 | 0.300 | 0.179 |  |  |  |  |  |
| Model 2^a^ | $\beta_{0}$ | 3.001 | 0.784 | 1.465 | 4.538 | <.001 | 717.011 | 0.879 | 0.835-0.923 | 0.880 | 0.877-0.882 |
|  | $\beta_{1}$ | 0.019 | 1.254 | -2.438 | 2.476 | 0.988 |  |  |  |  |  |
|  | $\beta_{2}$ | -0.163 | 0.023 | -0.208 | -0.118 | <.001 |  |  |  |  |  |
|  | $\beta_{3}$ | -0.014 | 0.038 | -0.089 | 0.061 | 0.720 |  |  |  |  |  |
| Model 3 | $\beta_{0}$ | 24.790 | 5.121 | 14.753 | 38.426 | <.001 | 677.330 | 0.894 | 0.854-0.934 | 0.894 | 0.892-0.896 |
|  | $\beta_{1}$ | 2.666 | 2.336 | -1.913 | 7.244 | 0.254 |  |  |  |  |  |
|  | $\beta_{2}$ | -4.987 | 1.017 | -6.980 | -2.995 | <.001 |  |  |  |  |  |
|  | $\beta_{3}$ | -0.294 | 0.163 | -0.615 | 0.026 | 0.071 |  |  |  |  |  |
|  | $\beta_{4}$ | 0.049 | 0.028 | -0.007 | 0.104 | 0.084 |  |  |  |  |  |
|  | $\beta_{5}$ | -0.577 | 0.455 | -1.469 | 0.315 | 0.205 |  |  |  |  |  |
| Model 4 | $\beta_{0}$ | 25.835 | 4.959 | 16.116 | 35.554 | <.001 | 678.33 | 0.895 | 0.855-0.935 | 0.895 | 0.893-0.897 |
|  | $\beta_{1}$ | 0.148 | 0.595 | -1.017 | 1.314 | 0.803 |  |  |  |  |  |
|  | $\beta_{2}$ | -5.222 | 0.984 | -7.150 | -3.295 | <.001 |  |  |  |  |  |
|  | $\beta_{3}$ | -0.293 | 0.163 | -0.613 | 0.026 | 0.072 |  |  |  |  |  |
|  | $\beta_{4}$ | 0.049 | 0.028 | -0.005 | 0.104 | 0.077 |  |  |  |  |  |
|  | $\beta_{5}$ | -0.014 | 0.018 | -0.049 | 0.021 | 0.433 |  |  |  |  |  |
| Model 5 | $\beta_{0}$ | 22.983 | 5.211 | 12.770 | 33.197 | <.001 | 677.789 | 0.897 | 0.858-0.936 | 0.897 | 0.895-0.899 |
|  | $\beta_{1}$ | 8.596 | 5.187 | -1.570 | 18.762 | 0.098 |  |  |  |  |  |
|  | $\beta_{2}$ | -4.465 | 1.074 | -6.571 | -2.359 | <.001 |  |  |  |  |  |
|  | $\beta_{3}$ | -0.338 | 0.165 | -0.660 | -0.015 | 0.041 |  |  |  |  |  |
|  | $\beta_{4}$ | 0.052 | 0.028 | -0.003 | 0.106 | 0.064 |  |  |  |  |  |
|  | $\beta_{5}$ | -2.144 | 1.307 | -4.705 | 0.416 | 0.101 |  |  |  |  |  |
|  | $\beta_{6}$ | 0.067 | 0.052 | -0.035 | 0.169 | 0.198 |  |  |  |  |  |

Coef, coefficient. Est, estimate. SE, standard error. 95% CI, 95% confidence interval. AIC, Akaike information criterion. ROC-AUC, area under the Receiver Operating Characteristic curve. GA, gestational age. BW, birth weight. DF, degree of freedom.

^a^ Scaling parameter for labeled models was set to 1 due to overdispersion (Pearson Chi-square/DF>2).

**List of Supplementary Figures**

**S1 Fig** Frequency distribution of included neonates in the US 2014-2018 birth cohort by sex.

A, female plot. B, male plot. BW, birth weight; GA, gestational age.

**S2 Fig** Observed moving sum of residuals plots for GA-based, BW-based, and BW-GA-based model for neonatal mortality rates among selected Pacific Islander neonates in the 2014-2018 US birth cohort.

A, GA-based model. B, BW-based model. C, BW-GA-based model. BW, birth weight; GA, gestational age. All models are adjusted by neonatal sex.

**S3 Fig** Decile calibration plot for non-bootstrapped data, models for GA-based, BW-based, and BW-GA-based model for neonatal mortality rates among selected Pacific Islander neonates in the 2014-2018 US birth cohort.

A, GA-based model. B, BW-based model. C, BW-GA-based model. BW, birth weight; GA, gestational age. All models are adjusted by neonatal sex.

**S4 Fig** Decile calibration plot for bootstrapped data, models for GA-only, BW-only, and BW-GA model for neonatal mortality rates among selected Pacific Islander neonates in the 2014-2018 US birth cohort.

A, GA-based model. B, BW-based model. C, BW-GA-based model. BW, birth weight; GA, gestational age. All models are adjusted by neonatal sex.

**S5 Fig** Observed moving sum of residuals plots for other explored Poisson regression models for neonatal mortality rates among selected Pacific Islander neonates in the 2014-2018 US birth cohort.

A, Model 1. B, Model 2. C, Model 3. D, Model 4. E, Model 5.

**S6 Fig** Decile calibration plot for non-bootstrapped data, other explored Poisson regression models for neonatal mortality rates among selected Pacific Islander neonates in the 2014-2018 US birth cohort.

A, Model 1. B, Model 2. C, Model 3. D, Model 4. E, Model 5.

**S7 Fig** Decile calibration plot for bootstrapped data, other explored Poisson regression models for neonatal mortality rates among selected Pacific Islander neonates in the 2014-2018 US birth cohort.

A, Model 1. B, Model 2. C, Model 3. D, Model 4. E, Model 5.
