# Supplementary figures and images for: Prediction of neonatal survival among Pacific Islander preterm births in the US
Source: PLoS One. 2024 Dec 31;19(12):e0316048. doi: 10.1371/journal.pone.0316048 (PMC11687717; doi:10.1371/journal.pone.0316048)

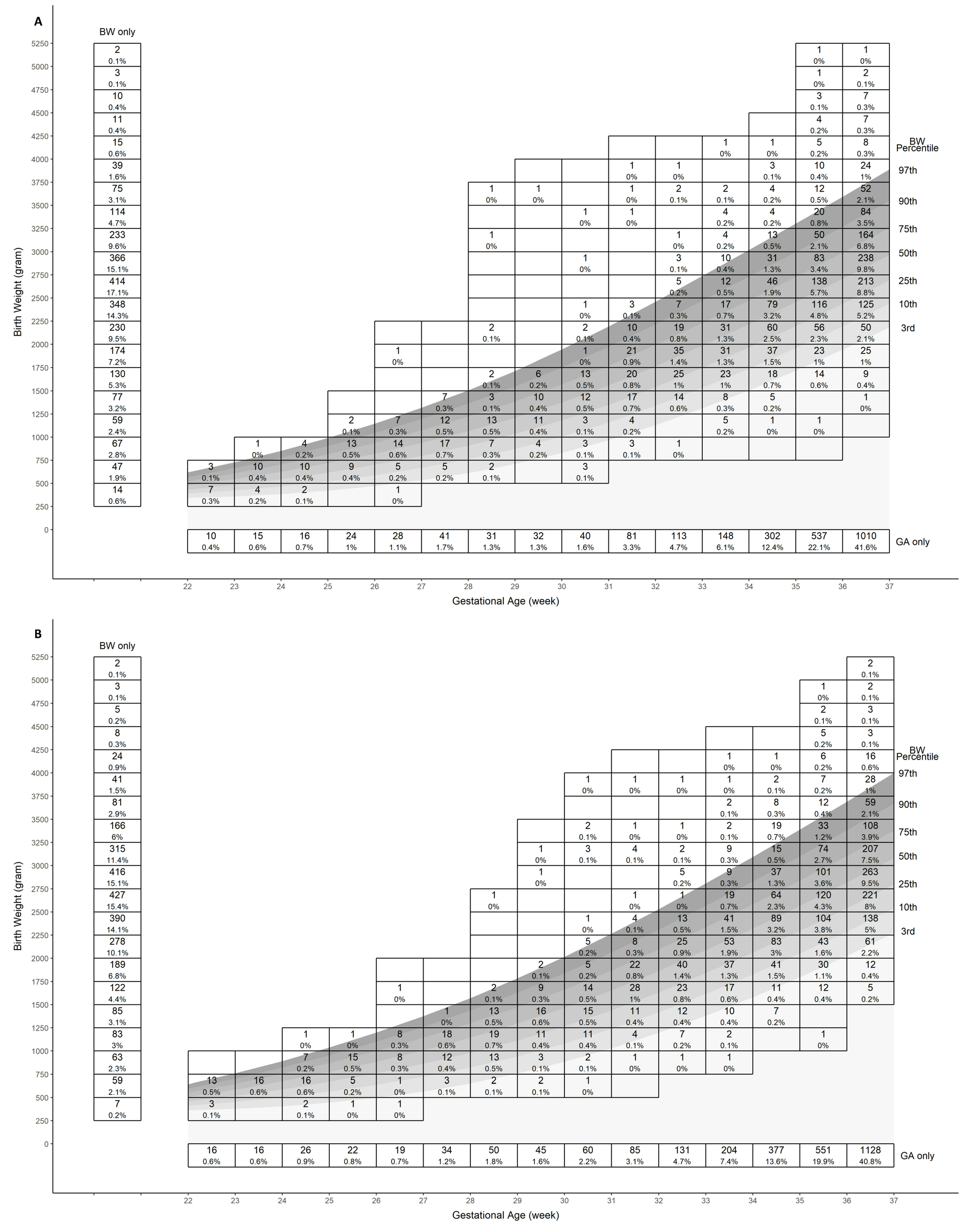

Supplement: S1 Fig — A, female plot. B, male plot. BW, birth weight; GA, gestational age. (TIF) [file pone.0316048.s002.tif]

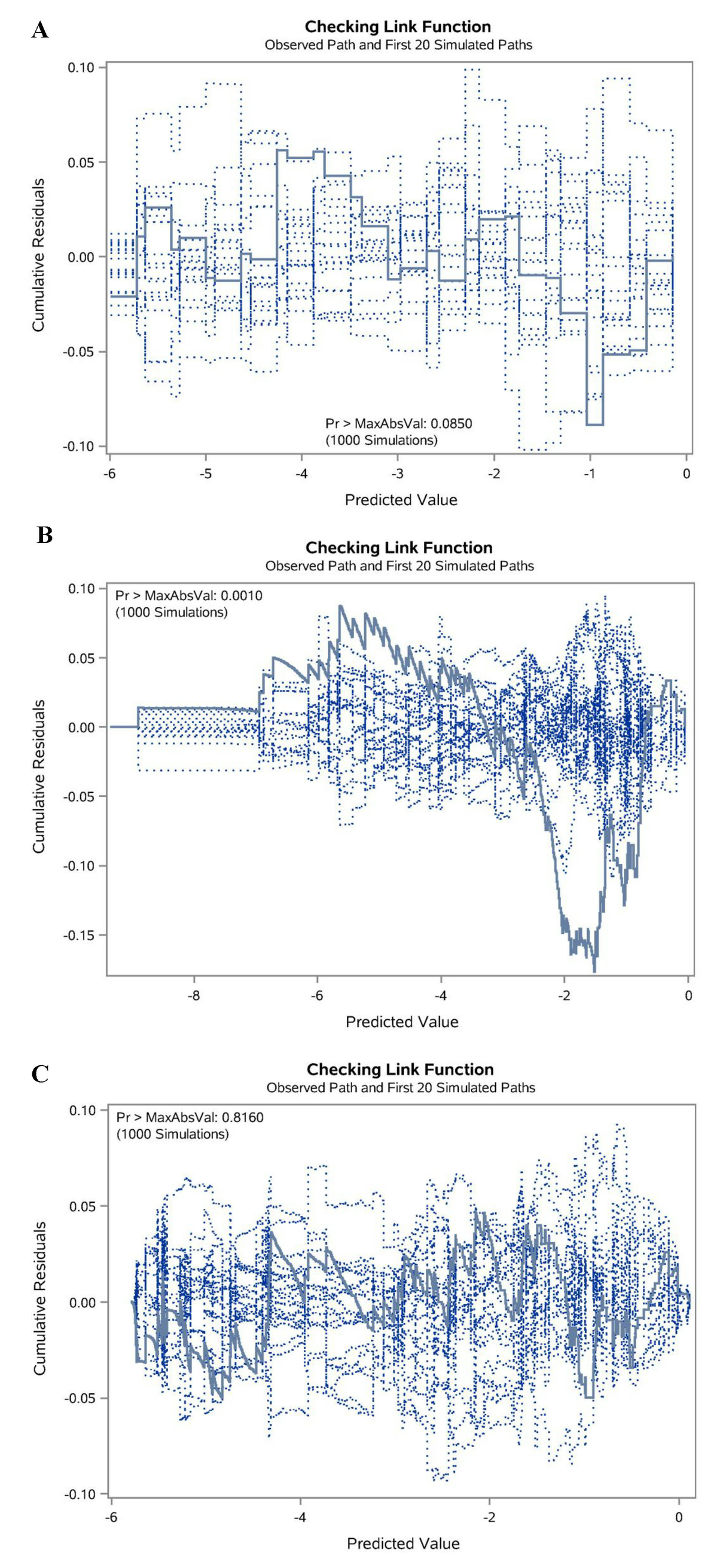

Supplement: S2 Fig — A, GA-based model. B, BW-based model. C, BW-GA-based model. BW, birth weight; GA, gestational age. All models are adjusted by neonatal sex. (TIF) [file pone.0316048.s003.tif]

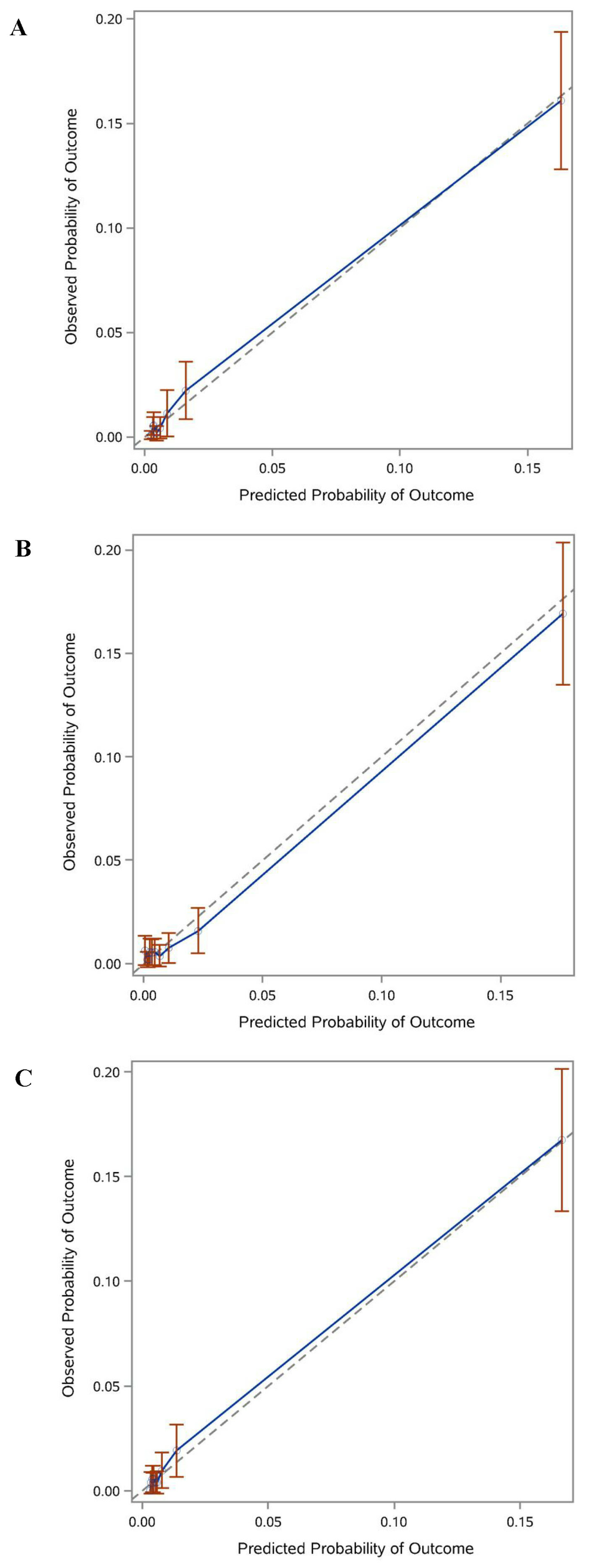

Supplement: S3 Fig — A, GA-based model. B, BW-based model. C, BW-GA-based model. BW, birth weight; GA, gestational age. All models are adjusted by neonatal sex. (TIF) [file pone.0316048.s004.tif]

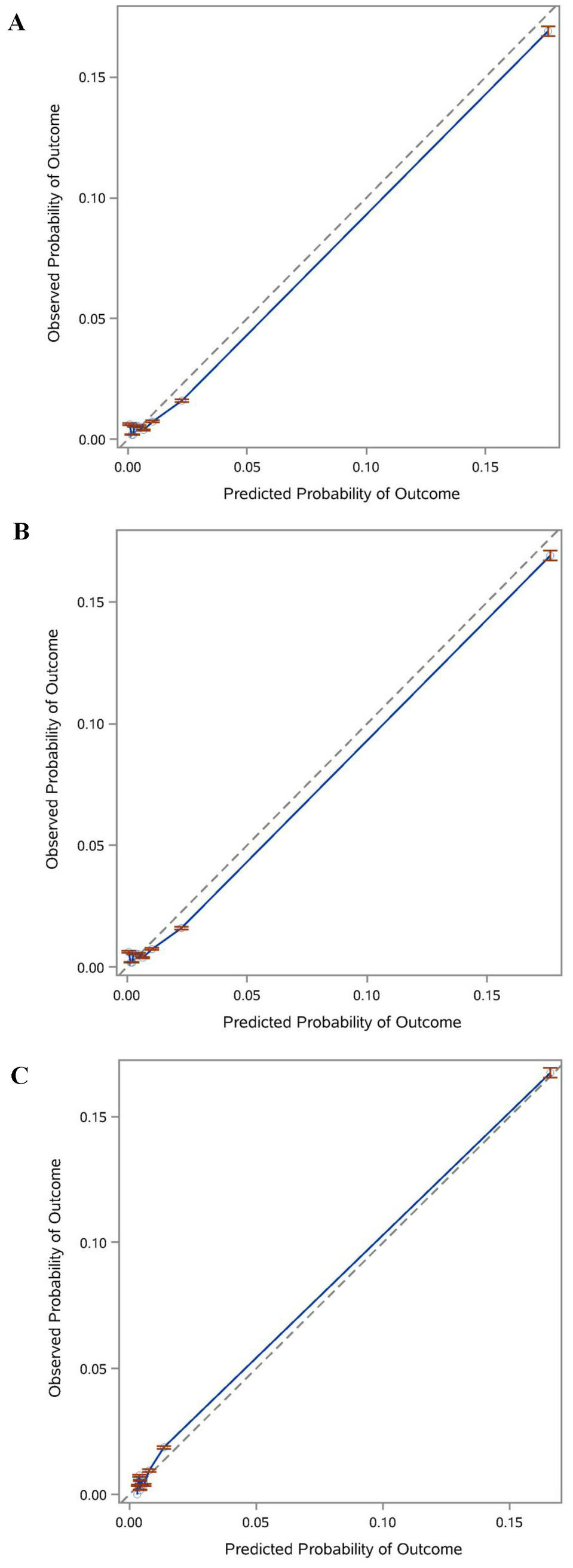

Supplement: S4 Fig — A, GA-based model. B, BW-based model. C, BW-GA-based model. BW, birth weight; GA, gestational age. All models are adjusted by neonatal sex. (TIF) [file pone.0316048.s005.tif]

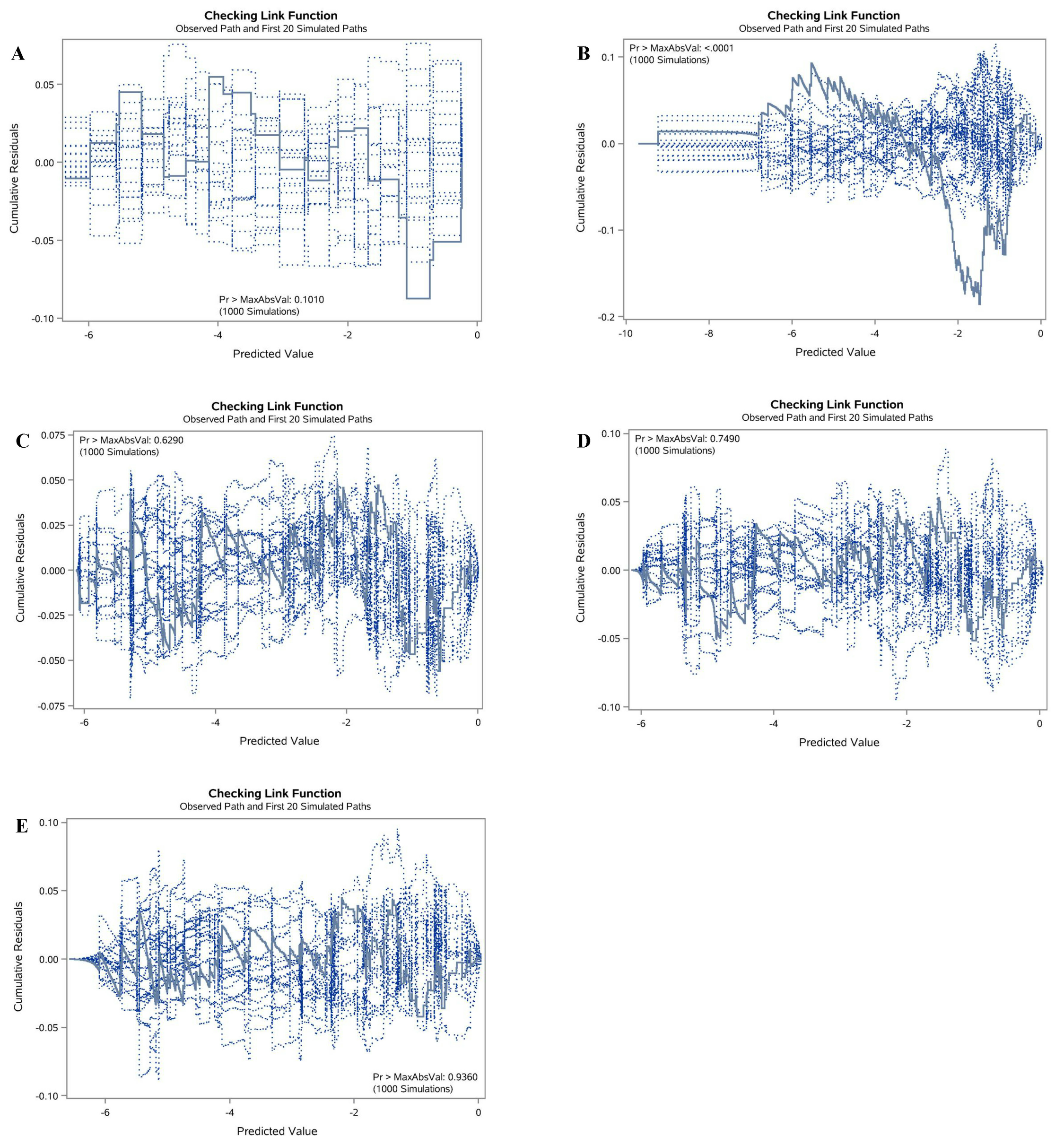

Supplement: S5 Fig — A, Model 1. B, Model 2. C, Model 3. D, Model 4. E, Model 5. (TIF) [file pone.0316048.s006.tif]

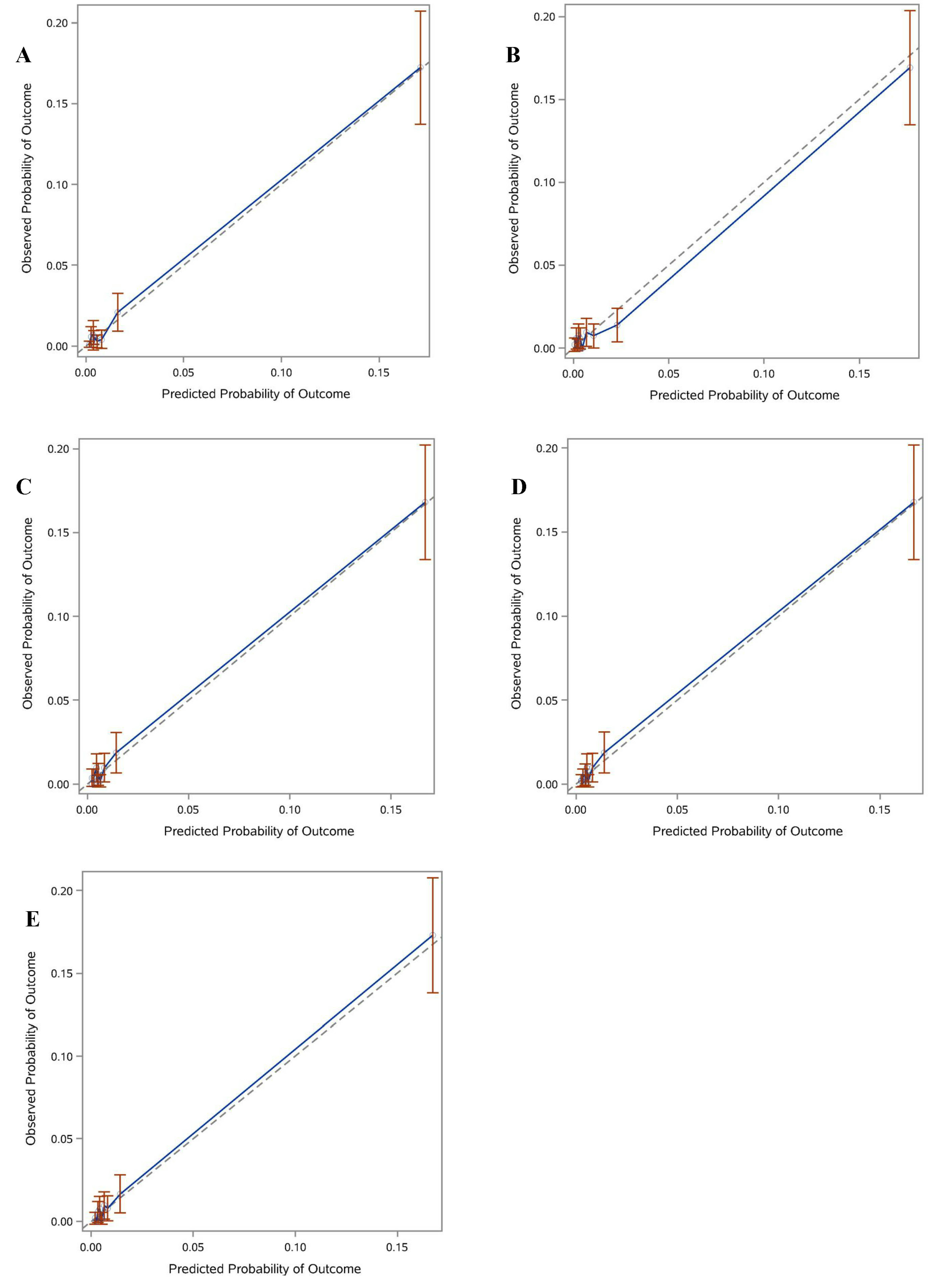

Supplement: S6 Fig — A, Model 1. B, Model 2. C, Model 3. D, Model 4. E, Model 5. (TIF) [file pone.0316048.s007.tif]

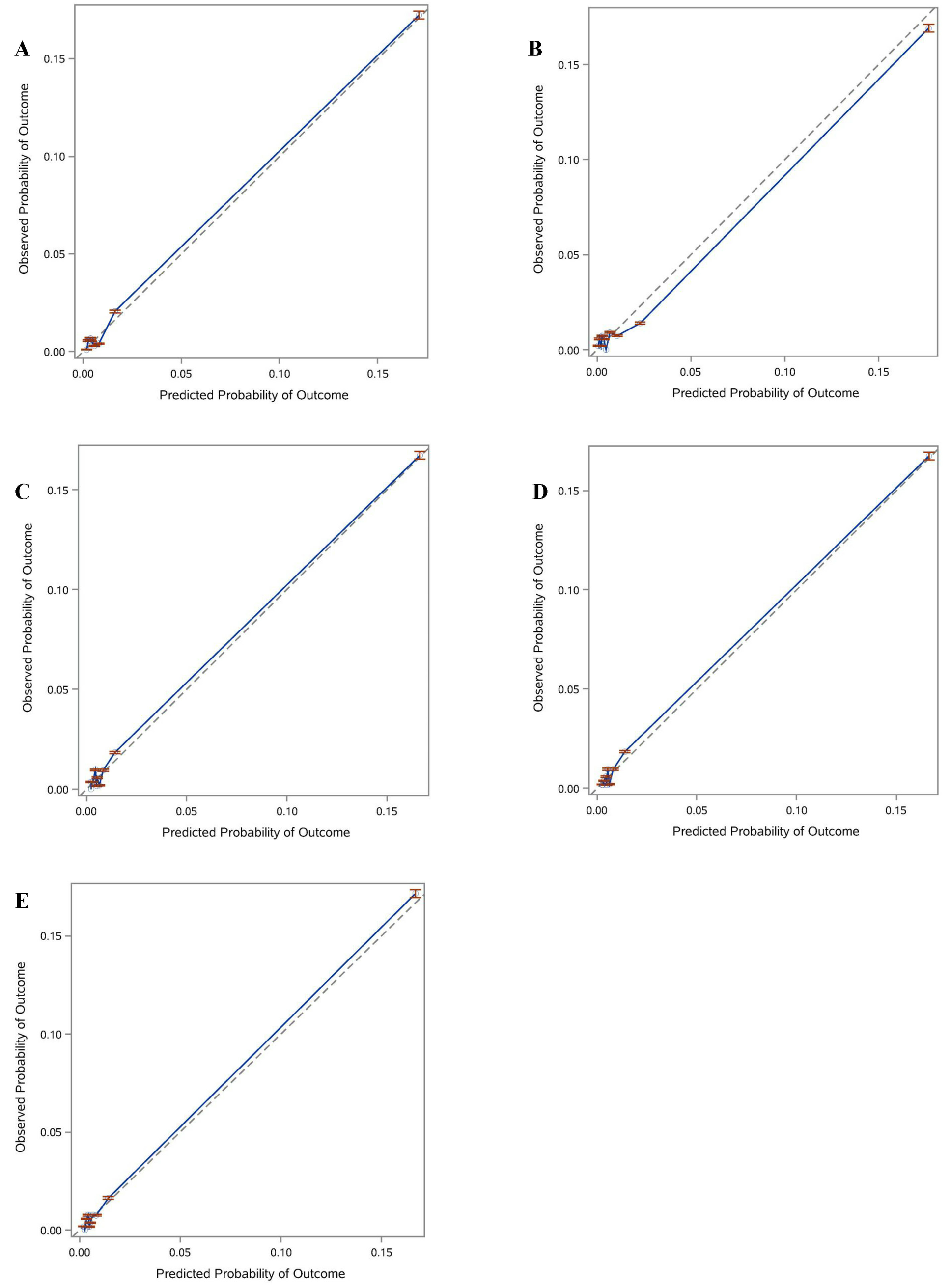

Supplement: S7 Fig — A, Model 1. B, Model 2. C, Model 3. D, Model 4. E, Model 5. (TIF) [file pone.0316048.s008.tif]
